# Multiple, Multiloculated, and Recurrent Keratocysts of the Mandible and Maxilla in Association with Gorlin-Goltz (Nevoid Basal-Cell Carcinoma) Syndrome: A Pediatric Case Report and Follow-up over 5 Years

**DOI:** 10.1155/2018/7594840

**Published:** 2018-09-19

**Authors:** P. Santander, E. M. C. Schwaibold, F. Bremmer, S. Batschkus, P. Kauffmann

**Affiliations:** ^1^Department of Orthodontics, University Medical Center, Göttingen, Germany; ^2^Institute of Human Genetics, University Medical Center, Göttingen, Germany; ^3^Institute of Pathology, University Medical Center, Göttingen, Germany; ^4^Department of Maxillofacial Surgery, University Medical Center, Göttingen, Germany

## Abstract

**Background:**

We report a case of multiple keratocysts first diagnosed in an 8-year-old boy.

**Case report:**

The incidental radiographic finding of a cystic lesion in an 8-year-old boy led to the surgical enucleation and further diagnosis of a keratocyst associated with a tooth crown. In the course of dental maturation from deciduous to permanent teeth, the boy presented new lesions, always associated with the crowns of teeth. Gorlin-Goltz (nevoid basal-cell carcinoma) syndrome was suspected, and the genetic analysis detected a previously undescribed germline variant in the *PTCH1* gene.

**Treatment:**

This included a surgical removal of the cystic lesions, as well as the affected teeth.

**Follow-up:**

Due to the high recurrence rate of the keratocysts, frequent radiological checks were performed over a 5-year period.

## 1. Introduction

Keratocysts are comparatively rare benign neoplasms. In 2005, this formerly designated odontogenic cyst was reclassified by the World Health Organization as a tumor [1] due to its local aggressive and recurrent behavior. This term “tumor,” however, has been somewhat controversial due to a lack of clearly described neoplastic etiology. In the new revision by the WHO in 2017 [2], the name of “odontogenic keratocyst” (OKC) was reinstituted [3]. OKCs are mostly solitary lesions that can radiologically present a single chamber or septations. Despite their heterogeneous features, OKCs appear most frequently in the 3^rd^ molar area of the mandible, in the 2–4^th^ decade of life, with a slight predominance in males [4, 5]. The nature and mainly the high recurrence of OKCs is still a matter of discussion. It has been proposed that difficult operative access, leaving affected teeth in place after treatment, cystic satellite formation, and the association with Gorlin-Goltz syndrome are correlated with a higher recurrence of OKCs [6, 7].

The “Gorlin-Goltz syndrome” has also been called “nevoid basal-cell carcinoma syndrome” or more descriptively “multiple basal epithelioma, jaw cysts, and bifid rib syndrome” [8, 9]. This syndrome is an autosomal dominant condition characterized by the presence of recurrent basal cell carcinoma and skeletal anomalies such as bifid ribs, OKCs, palmar and plantar pits, and other heterogeneous symptoms which often make diagnosis difficult [10–12]. Pathogenic heterozygous variants in the genes *PTCH1*, *PTCH2*, and *SUFU* are associated with Gorlin-Goltz syndrome. Apart from Gorlin-Goltz syndrome, the presence of multiple keratocystic lesions is exceptionally rare. According to the first international colloquium on nevoid basal-cell nevus syndrome [13] and to Evans et al. [14], the diagnosis of a Gorlin-Goltz syndrome should be suspected when (a) one major criteria and molecular confirmation, (b) two major criteria, or (c) one major and two minor criteria are found as listed in Table 1.

Due to the high recurrence rate of the OKCs and the consequences of the mostly extensive lesions, different therapeutic approaches and control schemes have been discussed [7]. In the following report, we present a pediatric male patient diagnosed with Gorlin-Goltz syndrome with heterogeneous, recurrent, and multiple OKCs. We describe the surgical management and the development over 5 years after the initial presentation.

## 2. Case Presentation

**Figure 1:**
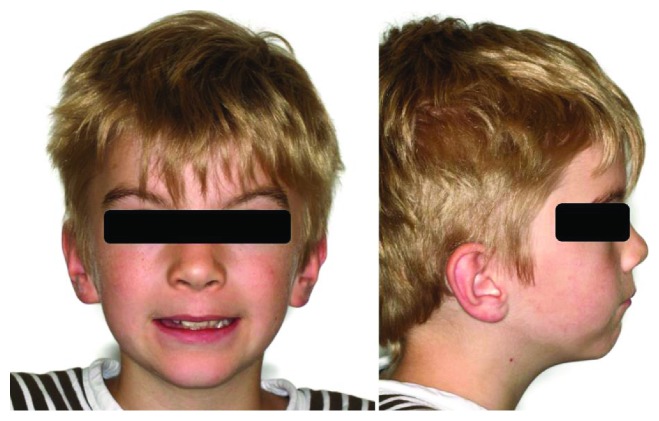
Extraoral photographs of the patient at first presentation in 2012.

**Figure 2:**
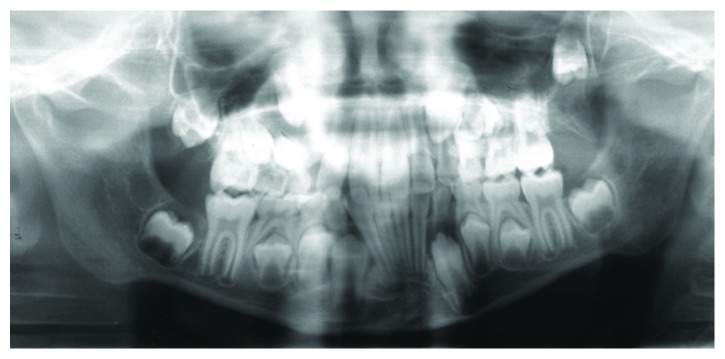
Panoramic radiograph (January 2012). Unclear radiolucency at teeth 13 and 23. Ectopic tooth 27.

The 8-year-old boy first presented in January 2012, having been referred by the family dentist, to the Department of Orthodontics at the Medical Center of the University of Göttingen for a routine orthodontic control and evaluation of treatment need (Figure 1). The clinical examination of the asymptomatic patient showed no extra- or intraoral pathological findings. The medical history of the boy included a mild pulmonary valve stenosis and a secundum atrial septal defect with a left-right shunt. He showed a good physical and cardiac fitness and a normal nutritional status. The family history was positive for maxillofacial anomalies: the boy's older sister had been previously diagnosed with a dysplastic fibroma, a rare benign fibrovascular defect in the mandible, and a resection of the affected area in the mandible had been performed. His father and paternal grandmother had a positive history of odontogenic cysts as well as basal cell carcinomas, although the family history of OKCs was negative. The radiological examination showed three suspicious hypomineralisations visible as radiolucencies in the panoramic radiograph associated with the retained teeth 13 and 23 and the ectopic tooth 27 (Figure 2). The young patient was referred to the Department of Maxillofacial Surgery for a surgical examination of the radiologic anomalies.

### 2.1. Treatment

**Figure 3:**
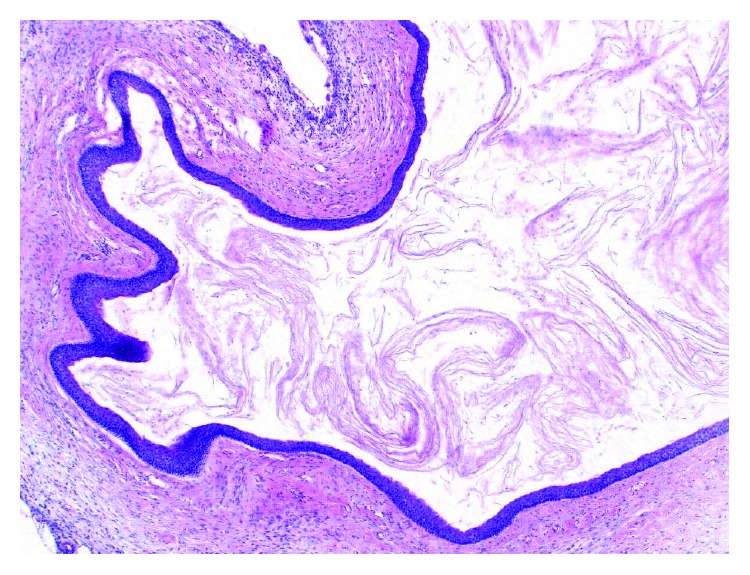
Histopathologic specimen (HE stain, 5x magnification).

**Figure 4:**
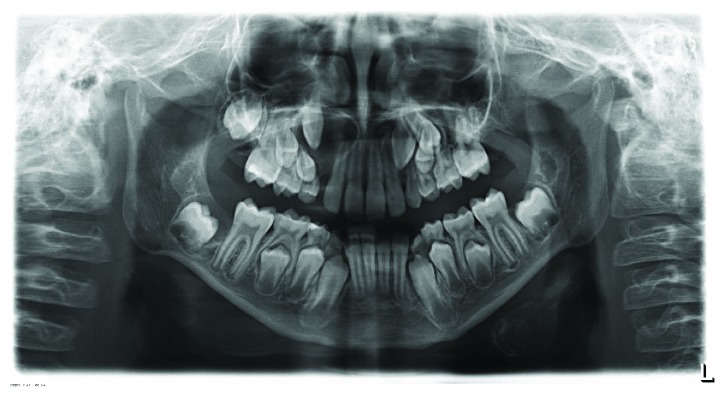
Panoramic radiograph (August 2012). 6 months after therapy, the radiolucent area in the region of tooth 23 was controlled. On the basis of the previous pathological findings, no further surgery was indicated.

The operation was performed under general anesthesia. The suspected pathological area around teeth 23 and 13 showed no visible intraoperative pathological signs. A bone and soft tissue biopsy for histological examination was taken. In the area of tooth 27, a well-marked membrane was revealed, filled with a viscid fluid and fully enclosing the dental crown. The clinical aspect was consistent with a follicular cyst. During the radical cystectomy, tooth 27 was removed due to massive attachment loss. The histopathological biopsy showed a fibroosseous lesion in the area of teeth 13 and 23. The biopsy from region 27 showed an odontogenic connective tissue cyst wall with intramural odontogenic cell islands. On request of the surgeon, samples were sent for further diagnosis to the Bone Tumor Reference Center of the Swiss Society of Pathology at the University Hospital in Basel, Switzerland. The initial histological diagnosis was corrected to an OKC of the parakeratin variant. Microscopically, the cyst shows a squamous epithelium. The basal cells are palisading, with hyperchromatic nuclei (HE staining, 5x magnification) (Figure 3). Due to the high recurrence of OKCs, a radiological control interval of 6 months was indicated (Figure 4). Furthermore, orthodontic treatment was initiated.

**Figure 5:**
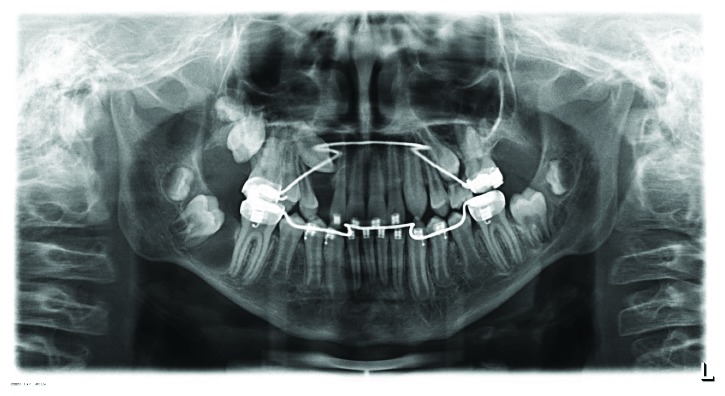
Panoramic radiograph (August 2014). Evaluation of the region of tooth 13. No manifest change and therefore no indication for further therapy. Radiolucency around the crown of the retained tooth 47, with indication for cystectomy.

In August 2014, during a regular radiological control, a new radiolucency was detected, associated with the retained and displaced teeth 47 and 48 (Figure 5). The surgical removal of the cystic lesion and tooth 47 was performed under general anesthesia. The pathological finding was consistent with an OKC.

**Figure 6:**
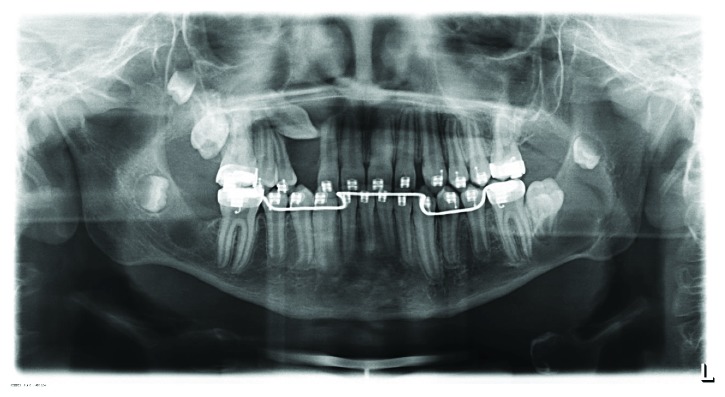
Follow-up panoramic radiograph (October 2015) shows a clear enlargement of the translucent area at tooth 13, as well as new changes associated to the crowns of teeth 18, 17, 37, 38, and 48.

**Figure 7:**
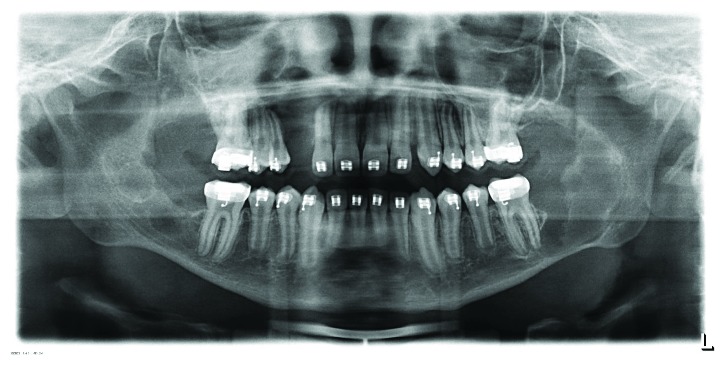
Postoperative control panoramic radiograph (October 2015).

The regular control examinations were interrupted by missed appointments, so the next evaluation took place one year later, in October 2015 (Figure 6). New radiolucencies were detected in the panoramic radiograph associated with the retained teeth 18, 17, 37, 38, and 48 as well as an evident enlargement of the radiolucency around the crown of tooth 13. A cone beam computer tomography scan was performed and showed well-defined radiolucent areas, associated with the retained teeth. Details of the surgical enucleation of the cysts with the extraction of teeth 18, 17, 13, 37, 38, and 48 are shown below. The postoperative radiological examination is depicted in Figure 7. Clinical and radiological examinations were then performed every 6 months.

### 2.2. Surgery

**Figure 8:**
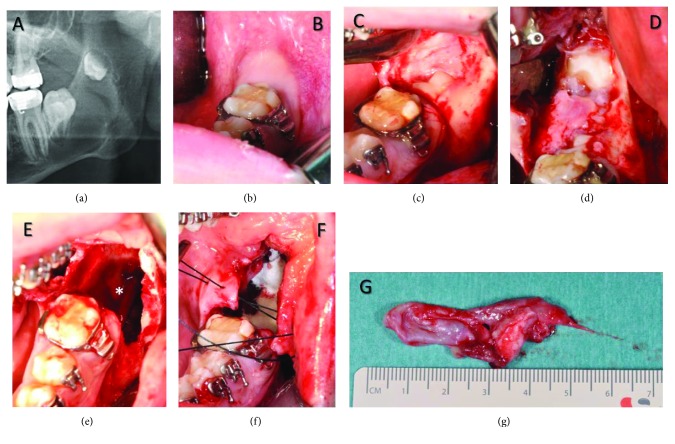
Enucleation of the cystic lesion. (a) Radiological finding of the affected area on the left side of the mandible. (b) Clinical situation, tooth 37 is not erupted. (c) Lifting of the mucoperiosteal flap, a perforation of the cortical bone is visible, as well as tooth 37. (d) Exposition of the displaced tooth 38. (e) Clinical situation after radical cystectomy. The lower alveolar nerve is intact and marked with an asterisk (^∗^). (f) Insertion of a collagen graft and suture. (g) Cystic lesion *in toto*.

We describe the surgical enucleation of the cystic lesions using the example of the third operation (2015). This was performed under general anesthesia; the affected regions were exposed after lifting a mucoperiosteal flap. After a careful removal of a thin bone cortex, the cystic capsule was found (Figure 8(a)) and separated from the bone with an obtuse instrument. The aim was to leave no epithelial remnants on the trabecular bone. All four lesions were associated with a retained tooth, which was only loosely anchored in the alveolar bone. Due to the high recurrence rate of the cystic lesions in this particular case, all affected teeth were extracted. In the area of the mandible, the use of Carnoy's solution was not indicated because of the exposure of the lower alveolar nerve (Figure 8(e)). Due to their large size, the cystic cavities were filled with a collagen graft, which stabilized the formation of a coagulum. No reconstruction with iliac crest bone or allogenic bone grafts was attempted. Subsequently, the mucoperiosteal flap was reverted back to its original position and fixed by sutures.

**Figure 9:**
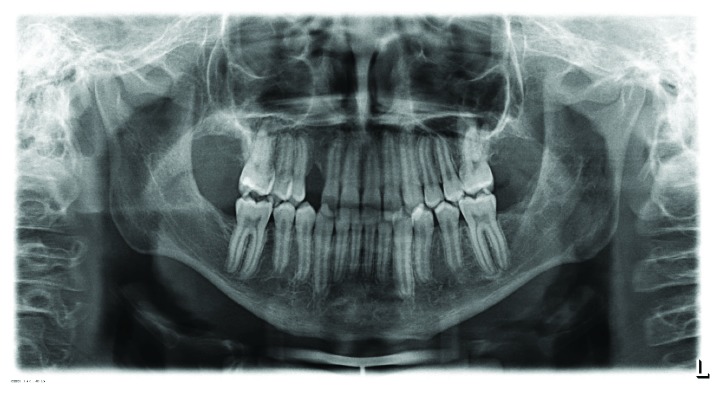
Panoramic radiograph (October 2016) 12 months after the third surgery.

**Figure 10:**
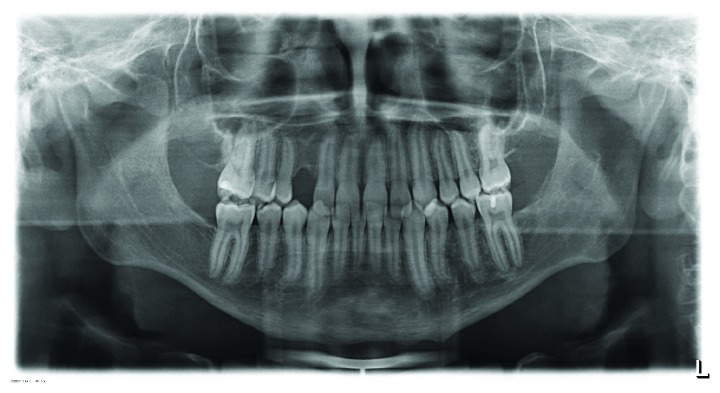
Follow-up panoramic radiograph (August 2017). Bone apposition in the former cystic cavities. No new lesions or recurrence is visible.

By October 2016 and August 2017, bone remodeling of the affected area had been detected and no new lesions were observed (Figures 9 and 10).

Due to the recurrence and the appearance of new lesions, Gorlin-Goltz syndrome was suspected in the patient. After genetic counselling at the Institute of Human Genetics of the University Medical Center of Göttingen, molecular genetic analysis of the genes *PTCH1* and *PTCH2* was performed in 2015. Sanger sequencing revealed the heterozygous germline variant c.2779_2793del (p.Ser927_Val931del) in the *PTCH1* gene. This variant leads to an “in-frame” deletion of 5 amino acids between amino acid positions 927 and 931 of the protein. This variant is listed neither in the Human Gene Mutation Database (HGMD) nor in the Leiden Open Variation Database (LOVD). However, a pathogenic effect of the variant seemed likely as many pathogenic variants have already been described in this region of the *PTCH1* gene, even several in-frame deletions [15, 16]. Since the boy's father had shown similar symptoms (odontogenic cysts, basal cell carcinomas) that could be in line with a Gorlin-Goltz syndrome, he, too, was tested for the *PTCH1* variant and resulted to be carrier of the variant.

In summary, clinical and molecular data together with the positive segregation analysis led to the classification of the variant as “probably pathogenic” and being responsible for Gorlin-Goltz syndrome in the patient and his father. The importance of talking precautions (e.g., sun protection due to the high risk of basal cell carcinomas) and regular medical surveillance (e.g., regular orthodontic care and annual dermatologic examinations) was emphasized.

## 3. Discussion

Gorlin-Goltz syndrome is an autosomal dominant inherited disorder resulting from pathogenic heterozygous germline variants in either of the genes *PTCH1*, *PTCH2*, and *SUFU*. It is relatively rare, and its phenotype can vary with more than 100 anomalies being associated with this syndrome. This diversity in clinical manifestations can lead to misdiagnoses and often to late diagnoses, associated with major bone defects. Hence, the experience of the practitioner, who correctly interprets the signs and draws a connection to a possibly underlying syndrome, and the experience of the pathologist, who is able to identify subtle histological differences, are essential [13].

According to the recommendations of Evans et al. [14] and Lo Muzio et al. [11], initially, no direct link to Gorlin-Goltz syndrome had been established in our patient since only one of the major criteria was fulfilled. The conspicuous repetitive appearance of the cystic lesions, which were all pathologically confirmed as OKCs, led to the recommendation of performing a genetic evaluation of the patient and his direct relatives. A positive family history of “odontogenic cysts” as well as basal cell carcinoma additionally pointed towards Gorlin-Goltz syndrome. As reported by Kulkarni et al. [17], only a few patients with multiple OKCs have other characteristic symptoms of Gorlin-Goltz syndrome. However, it has been suggested that multiple keratocysts alone may be confirmatory of this syndrome.

According to Casaroto et al. [18], OKCs are often the first manifestation of Gorlin-Goltz syndrome in children. Nevertheless, and as mentioned by Lo Muzio et al. [11], Gorlin-Goltz syndrome should be suspected in pediatric patients under 10 years of age presenting with OKCs at this early age. Reinforcing this recommendation, the study group of Ahn et al. [19] pointed out that 90% of the Gorlin-Goltz syndrome patients presented with OKCs.

The boy's presentation with bilateral or more than three lesions at the same time, which were mainly localized in the posterior region of the mandible and associated with retained teeth, even causing tooth or germ displacement, is also highly associated with Gorlin-Goltz syndrome [20]. This conclusion of Gupta et al. [20] is consistent with the manifestations in the boy. A substantial difference was observed regarding the occurrence of new lesions, since Gupta et al. [20] described that there were no recurrences or new manifestations after an observation period of 11–20 months. This should not be confused with a reoccurrence of the initial lesion. Reoccurrence of OKCs is frequent, ranging from 2.5% to 62%, as reported by Bell and Dierks [21]. As presented in our patient, reoccurrence as well as new lesions can be observed, especially as a manifestation for Gorlin-Goltz syndrome.

Despite not being characteristic of Gorlin-Goltz syndrome, 40% of the OKCs are associated with retained teeth, as mentioned by Díaz-Belenguer et al. [22]. This is a property that makes a differential diagnosis to follicular cysts difficult. Our patient was remarkable for the relation of the cystic lesions and the tooth crown, as all lesions were associated with retained teeth and involved the tooth crown.

## 4. Conclusions

The suspected diagnosis of an OKC is possible with radiological images and should be confirmed histopathologically. Due to the high recurrence risk, regular radiological follow-ups are important for treatment success. A possible presence of Gorlin-Goltz syndrome should be suspected in case of multilocular cysts in pediatric patients, even if further signs of the syndrome are not discernible at first sight and all pediatric patients with OKC diagnosis should be followed into adulthood. A genetic analysis confirms the diagnosis. In our patient, it led to the detection of a new, probably pathogenic germline variant in the *PTCH1* gene.

## Figures and Tables

**Table 1:** Diagnostic criteria for nevoid basal-cell carcinoma syndrome. A diagnosis can be made when two major or one major and two minor criteria are fulfilled. Extracted from Evans et al. [14].

Major criteria	Minor criteria
More than two basal cell carcinomas, or BCCs; one BCC at younger than 30 years of age; or more than 10 basal cell nevi	Congenital skeletal anomaly: bifid, fused, splayed or missing rib or bifid, wedged or fused vertebra
Any odontogenic keratocyst (proven on histology) or polyostotic bone cyst	Occipital-frontal circumference, more than 97 percentile, with frontal bossing
Three or more palmar or plantar pits	Cardiac or ovarian fibroma
Ectopic calcification: lamellar or early—at younger than 20 years of age—falx calcification	Medulloblastoma
Positive family history of nevoid basal-cell carcinoma syndrome	Lymphomesenteric cysts
	Congenital malformation such as cleft lip or palate, polydactylism, or eye anomaly (cataract, coloboma, microphthalmos)
